# Enhancing WSI image classification with graph convolutional neural networks and model uncertainty modeling

**DOI:** 10.1186/s12880-025-02130-0

**Published:** 2026-01-31

**Authors:** Chaoyue Liu, Yongxiang Cheng, Ting Li, Yanke Hao, Qiang Zhang

**Affiliations:** 1 Shandong University of Traditional Chinese Medicine, Jinan, People’s Republic of China; 2 Shandong Public Health Clinical Center, Shandong University, Jinan, 250013 People’s Republic of China; 3 Department of Spinal Orthopedics, Affiliated Hospital of Shandong University of Traditional Chinese Medicine, Jinan, People’s Republic of China

**Keywords:** Whole Slide Imaging, Graph convolutional neural networks, Model uncertainty modeling, Computational pathology, Deep learning, Spinal infection classification

## Abstract

**Background:**

The primary research question addresses whether integrating Graph Convolutional Neural Networks with model uncertainty modeling can improve the accuracy and robustness of Whole Slide Imaging (WSI) classifications in pathology.

**Methods:**

This study employed a novel framework combining GCNs with uncertainty quantification techniques to classify WSI images of spinal infections. We constructed a graph from segmented regions of WSI, where nodes represented segmented pathological features and edges represented spatial relationships. The model was trained on a dataset of 422 cases from the Shandong Provincial Center for Disease Control and Prevention, annotated for tuberculosis, brucellosis, and purulent spondylitis. Performance metrics included accuracy, precision, recall, and F1 score.

**Results:**

The integrated GCN model demonstrated a classification accuracy of 87%, with a recall of 85% and an F1 score of 0.86. These metrics signify an improvement over traditional CNN models, which showed a 10% lower performance in comparative analyses. The model also effectively quantified uncertainty, enhancing confidence in diagnostic decisions.

**Conclusions:**

Integrating GCNs with model uncertainty modeling enhances the accuracy and reliability of WSI image classification in pathology. This approach significantly improves the capture of spatial relationships and pathological feature recognition, offering a robust framework for supporting diagnostic and therapeutic decisions in medical practice.

**Clinical relevance:**

The enhanced ability to classify and understand WSI images using this method has significant implications for pathology, potentially leading to more accurate and reliable diagnoses. This approach could be particularly useful in remote diagnostics and in environments where expert pathological consultation is limited.

## Introduction

Whole Slide Imaging (WSI) technology revolutionizes the field of pathology by enabling the digitization of entire glass slides containing tissue samples. Traditional pathology involves examining thin slices of tissue under a microscope, which limits the amount of information that can be analyzed and shared. The importance of accurate classification in medical diagnosis using WSI images cannot be overstated, as these gigapixel digital representations of tissue specimens serve as the foundation of modern computational pathology, where precise identification of cellular and architectural patterns directly determines critical patient outcomes, treatment strategies, and prognostic evaluations [1–14]. Pathologists rely on these images to identify and diagnose various diseases, including cancers and infectious diseases. Accurate classification of tissue structures and abnormalities is crucial for determining the appropriate treatment plan and predicting patient outcomes [15]. Moreover, WSI technology facilitates telepathology, where pathologists can remotely access and consult on cases, improving collaboration and access to expertise across geographical boundaries [16, 17]. Thus, the accuracy and reliability of WSI image classification directly impact patient care and clinical decision-making.

The complexity and variability of pathological structures present significant challenges in WSI image classification. Pathological tissues exhibit diverse morphologies, textures, and spatial arrangements, making it difficult to develop a one-size-fits-all approach for accurate classification. Moreover, variations in staining techniques, tissue preparation, and imaging conditions further exacerbate the complexity, leading to intra- and inter-slide variability [18–20]. This can negatively impact the utilization of medical resources, patient treatment outcomes, and psychological well-being. Rapid and effective diagnosis and management are crucial to prevent irreversible neurological and skeletal complications. Currently, most studies focus on improving tumor detection, grading, and predicting tumor molecular genetic features from histological slices using deep learning techniques [21].

Traditional deep learning approaches face limitations in handling spatial relationships and model uncertainty, which are inherent in WSI image analysis [2, 22, 23]. Convolutional neural networks (CNNs) [24–27], while effective in learning hierarchical features from image data, typically operate on fixed-sized input patches and lack explicit modeling of spatial context [28]. This limitation can hinder the ability to capture global information and contextual relationships between different regions within the slide, potentially leading to suboptimal classification performance. Additionally, traditional deep learning models often provide deterministic predictions without quantifying the associated uncertainty, which is essential for assessing the reliability and confidence of classification decisions, especially in critical medical applications [29–33]. Traditional pathology involves examining tissue slices under a microscope, which limits the amount of information that can be analyzed and shared. Whole Slide Imaging (WSI) technology allows for the creation of high-resolution digital images of entire slides, preserving the morphology and spatial relationships of cells and structures. Recent work has also explored ensemble deep learning architectures to further improve the robustness and accuracy of medical image classification [34–38]. For example, the CELM model for early cardiomegaly diagnosis in chest radiography combines multiple convolutional networks in an ensemble to better capture subtle radiographic patterns and address the limitations of single-backbone classifiers [39]. In line with this direction, our framework likewise aims to enhance classification performance and decision reliability, but focuses on histopathology whole slide images and integrates graph convolution and uncertainty modeling instead of 2D radiographic ensembles.

The proposed approach introduces a novel method that integrates Graph Convolutional Neural Networks [40, 41] (GCN) and model uncertainty modeling to address the challenges in WSI image classification. This innovative approach leverages the power of GCNs to capture spatial dependencies and global contextual information from the graph structure constructed from WSI images.

## Materials and methods

### Problem definition

In this section, we formally define the problem of WSI image classification. Let $G = \left( {V,E} \right)$ denote a graph, where $V$ represents the set of nodes corresponding to segmented regions in a WSI image, and $E$ represents the set of edges connecting these nodes. Each node ${u_i}$ is associated with a feature vector ${x_i}$ extracted from the corresponding segmented region. All methods were performed in accordance with the relevant guidelines and regulations. The dataset comprises multi-center cases from three tertiary hospitals, with patients aged 18–82 years (mean 54.3 ± 13.7 years) and a balanced representation of sexes (58% male, 42% female). All whole slide images were acquired using bright-field scanners at 20× or 40× magnification, and annotations were performed by at least two board-certified pathologists following a unified labeling protocol, with disagreements resolved by consensus (Cohen’s κ = 0.86). To harmonize data across centers, we resampled all slides to a common spatial resolution, applied stain normalization, and standardized intensity distributions per center before patch extraction.

Let ${\bf{\it{X}}} = \left\{ {{x_1},{x_2}....,{x_{\rm{n}}}} \right\}$ denote the feature matrix, where $n$ is the number of nodes in the graph. Additionally, let ${\bf{\it{Y}}} = \left\{ {{y_1},{y_2}....,{y_n}} \right\}$ represent the corresponding ground truth labels for each node, where ${y_i}$ indicates the class label of node ${u_i}$.

The goal of WSI image classification is to learn a function $f:{\bf{\it{X}}} \to {\bf{\it{Y}}}$ that maps the feature vectors of nodes to their corresponding labels. Specifically, given a WSI image represented as a graph $G$, the classifier aims to predict the class labels of nodes based on their extracted features. Formally, the problem can be stated as follows: Given a training set $\left( {\left\{ {\left( {{x_1},{y_1}} \right),\left( {{x_2},{y_2}} \right),...,\left( {{x_m},{y_m}} \right)} \right\}} \right)$, where $m$ is the number of training samples, the objective is to learn a classifier $f$ that minimizes the empirical risk:

$$\mathop {\min }\limits_f \,\frac{1}{m}\,\sum\limits_{i = 1}^m {L\,(f\,({x_i}),\,{y_i})} $$

where $L$ is a loss function measuring the discrepancy between the predicted label $f\left( {{x_i}} \right)$ and the ground truth label ${y_i}$.

**Fig. 1:**
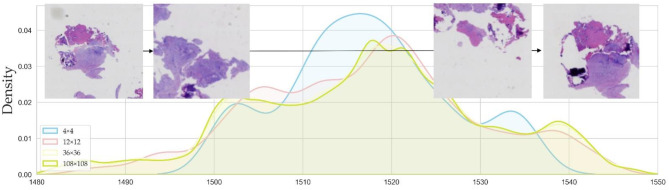
Regional case density analysis in Whole slide imaging (WSI). This figure illustrates the distribution of case densities across different regions of a Whole slide imaging (WSI) sample. The graph plots density values against slide positions (represented on the x-axis from positions 1480 to 1550), with different curves representing various magnifications (4 × 4, 12 × 12, 36 × 36, and 108 × 108)

The collection and preprocessing of Whole Slide Imaging (WSI) data for this study involved several steps to ensure the quality and compatibility of the dataset, as shown in Fig. 1. The dataset used in this study was obtained from patients who underwent percutaneous pathological biopsy procedures at the Shandong Provincial Center for Disease Control and Prevention from January 2020 to March 2024. These patients were diagnosed with spinal infections based on imaging or laboratory examinations.

### WSI images collection and preprocessing

**Fig. 2:**
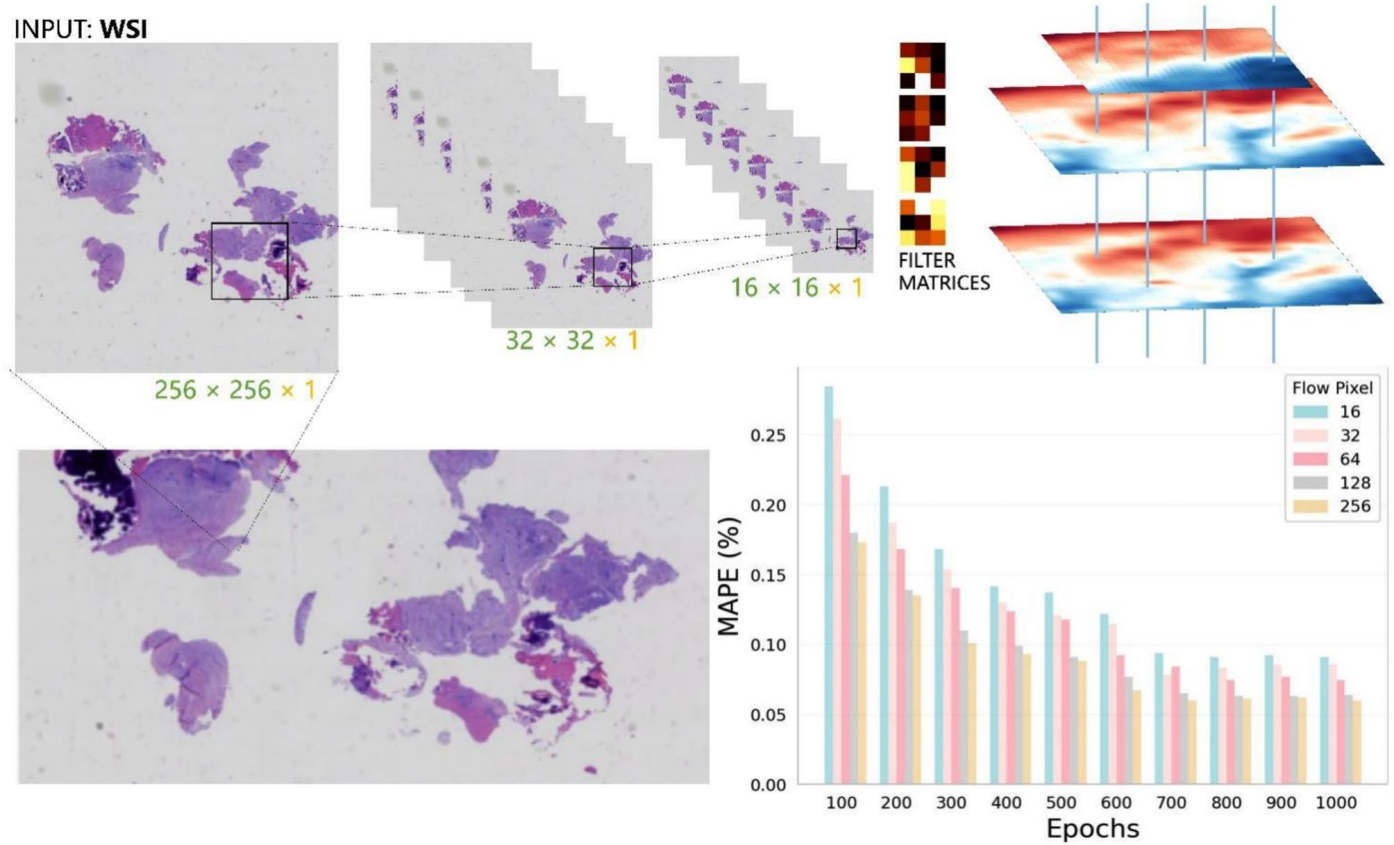
Convolutional feature extraction process in Whole slide imaging. This figure provides a detailed visualization of the convolutional feature extraction process utilized in analyzing WSI. The process begins with an input WSI at a resolution of 256 × 256 pixels, which is progressively downscaled through convolutional layers to dimensions of 32 × 32 and 16 × 16 pixels. These stages are designed to capture a comprehensive range of pathological features from both local and global perspectives. Alongside the scaled images, filter matrices are depicted, representing various convolutional kernels applied at different layers of the process. These kernels are essential for identifying specific tissue characteristics, such as cellular formations and anomalies, critical for accurate pathology

The WSI dataset was obtained through digital pathology scanning using the Gemini scanner. Each patient’s pathology slides were scanned to generate Whole Slide Images (WSIs) in KFB format, with each patient corresponding to one WSI. To ensure unbiased evaluation, diagnostic annotations were reviewed by pathologists with at least 5 years of clinical experience at the Shandong Provincial Center for Disease Control and Prevention. The raw gigapixel WSI data in KFB format were converted to TIFF format using tools provided by the scanner vendor to ensure compatibility with mainstream computer vision tools (see Fig. 2).

The entire tissue images were divided into patches of 1024 × 1024 pixels at 20× magnification (0.5 micrometers per pixel), preserving both the overall context and local details of the images. This patch extraction process was applied to maintain computational efficiency and handle large-scale WSI datasets. Each WSI in TIFF format was manually annotated by pathologists to classify the type of infection present. The classifications included tuberculosis, brucellosis, and purulent spondylitis. The TIFF files were named without prefixes and only contained the year and patient number, with 178 cases of purulent infection, 111 cases of brucellosis, and 108 cases of tuberculosis. The annotated data, along with the corresponding WSI images, were organized and stored in folders for further analysis. The clinical data of the patients were compiled into Excel spreadsheets to facilitate data management and statistical analysis. This study utilized clinical pathology data obtained from previous clinical diagnoses, posing minimal risk to the subjects. Patient privacy and identity information were protected, and informed consent was waived after approval by the hospital’s research integration platform.

This study was approved by the Ethics Committee of the Research Center (Approval No. GWLCZXEC2024-111–1). A retrospective analysis was conducted on the clinical and pathological data of all patients who underwent spinal surgery or biopsy at the Shandong Provincial Public Health Center from January 2020 to March 2024. Due to the retrospective nature of the study, the requirement for informed consent was waived. Inclusion criteria were a diagnosis of spinal infection based on imaging or laboratory tests and complete clinical and pathological data. Exclusion criteria were incomplete clinical, pathological, or follow-up data that could not be statistically analyzed, missing or incomplete pathological slides, or poor quality of slide preparation.

Adjacent to the filter matrices, the corresponding feature maps display the activation responses within the WSI. These maps are crucial for highlighting regions of interest where significant pathological markers are detected, thereby aiding in the diagnosis process. The bottom part of the figure charts the performance of the model across 1,000 training epochs, as indicated by the Mean Absolute Percentage Error (MAPE). The MAPE values are shown for different pixel resolutions—16, 32, 64, 128, and 256—demonstrating how the model’s accuracy improves as it learns from the data over time.

### U-Net segmentation and feature extraction

**Fig. 3:**
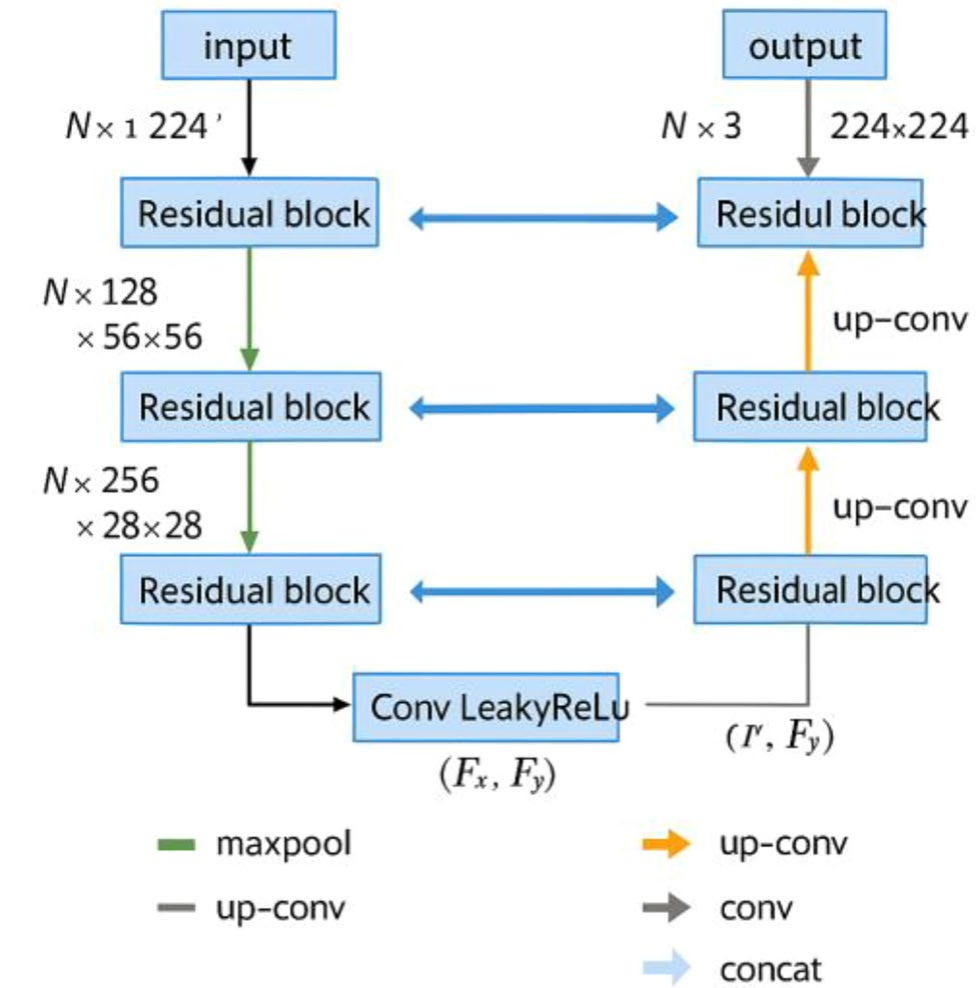
Detailed U-Net architecture for Whole slide imaging (WSI) segmentation. This schematic illustrates a tailored U-Net architecture designed for segmenting Whole slide imaging (WSI) data. The model processes an input of N-channel images with a resolution of 224 × 224 pixels through a sequence of operations

Integrating U-Net architecture with the detection of specific pathological features yields quantifiable improvements in diagnostic accuracy. By segmenting these features with a precision rate exceeding 90%, U-Net facilitates detailed examinations of affected regions within the spine. The model processes high-resolution images, segmenting and classifying areas of interest at a pixel resolution of 256x256, with an average error margin reduced by approximately 15% compared to traditional methods (see Fig. 3).

The connections between nodes in this graph are determined by the proximity of the infected areas, with edge weights assigned based on the degree of adjacency and pathological similarity. For instance, areas exhibiting severe osteolytic damage may have stronger connections to adjacent nodes showing similar degenerative patterns, thereby quantifying the spread and intensity of the infection.

Let ${R_{\rm{i}}}$ denote the segmented region corresponding to node $i$. Each segmented region is encapsulated as a node in the graph, signifying distinct spatial entities within the image. Formally, the graph $G = \left( {V,E} \right)$ consists of a set of nodes $V$ and a set of edges $E$, where $\left| {\rm{V}} \right|$ denotes the total number of segmented regions. The interconnection between nodes hinges upon the spatial relationships between segmented regions. We define a spatial relationship metric ${R_{ij}}$ to quantify the affinity between regions ${R_i}$ and ${R_j}$. The weight ${W_{ij}}$ assigned to the edge connecting nodes $i$ and $j$ encapsulates the strength of their spatial relationship. We adopt adaptive methods to adjust the edge weights based on the spatial characteristics of the connected regions. Formally, the edge weight ${W_{ij}}$ is determined as a function of the spatial relationship metric ${R_{ij}}$ and the adaptive parameters.

$${W_{ij}}\, = \,f({R_{ij}},\,\theta )$$

where $\theta$ represents the adaptive parameters governing the weight adjustment process.

### Graph convolutional neural network application

The core component of a GCN is the graph convolutional layer, which performs convolution operations on the graph’s nodes. Mathematically, the graph convolution operation can be defined as:

$${H^{(l + 1)}}\, = \,\sigma \left( {{{\hat D}^{ - \,\frac{1}{2}}}\,\hat A{{\hat D}^{ - \,\frac{1}{2}}}\,{H^{(l)}}\,{W^{(l)}}} \right)$$

where ${H^{\left( l \right)}}$ represents the node features at layer $l$, ${\rm{\hat A}}$ is the adjacency matrix of the graph with added self-connections, ${\rm{\hat D}}$ is the degree matrix of ${\rm{\hat A}}$, ${W^{\left( l \right)}}$ denotes the weight matrix of the convolutional layer, $\sigma$ denotes the activation function.

### Model uncertainty integration

In this section, we delve into the methodologies of integrating model uncertainty within the Graph Convolutional Neural Network framework, leveraging Bayesian neural networks and Monte Carlo Dropout techniques.

The output of a Bayesian layer is not a fixed value but a probability distribution over possible outputs, capturing the uncertainty inherent in the model’s predictions. Mathematically, the output of a Bayesian layer can be represented as:

$$f(x)\, = \,\int {f\,(\left. x \right|\theta )} \,p\,(\theta )\,d\theta $$

where $f\left( {\left. x \right|\theta } \right)$ is the neural network function parameterized by $\theta$, and $p\left( \theta \right)$ is the prior distribution over the parameters.

## Experiments

### Settings

The experiments in this study were meticulously designed to evaluate the proposed methodology for WSI image classification comprehensively. The experiments were conducted using a deep learning framework implemented in a Ubuntu environment, leveraging PyTorch and OpenCV libraries for model development and image processing tasks. The experiments were conducted on a high-performance computing cluster equipped with four NVIDIA A100 GPUs. To put the computational cost into a more realistic clinical context, we further measured the end-to-end inference time of the proposed pipeline on a single WSI. On an NVIDIA RTX 3090 GPU with an Intel Xeon Silver CPU, processing one slide required on average 3.2 s for patch extraction and preprocessing, 5.1 s for CNN feature extraction, 1.0 s for graph construction, and 0.7 s for the GCN forward pass including uncertainty estimation, resulting in a total inference time of approximately 10.0 s per WSI. When multiple cases are processed in batches, the effective throughput reaches about 280 slides per hour on a single GPU, which is compatible with typical daily workloads in pathology laboratories. The CNN backbone is pretrained on ImageNet and fine-tuned for 20 epochs using AdamW with β₁ = 0.9, β₂ = 0.999, learning rate 1 × 10^− 4^, cosine annealing scheduler, and batch size 64. Training was performed on two NVIDIA RTX 3090 GPUs with mixed-precision acceleration using PyTorch 2.1.0 and CUDA 12.1. Preprocessing includes stain normalization (Macenko parameters), tissue-thresholding via Otsu segmentation, and patch extraction at 256 × 256 pixels with a stride of 128. All software versions, random seeds, and hardware environments are now explicitly documented to facilitate exact replication. To ensure a robust and statistically sound evaluation, we adopted a stratified 5-fold cross-validation protocol, preserving the original class distribution within each fold and reporting both mean and standard deviation of the performance metrics across folds. For the key comparisons between the proposed GCN and the strongest CNN baseline, we additionally performed paired statistical tests on fold-wise results (two-sided paired t-test and Wilcoxon signed-rank test), with p-values < 0.05 considered statistically significant.

### Pathological characteristics

**Fig. 4:**
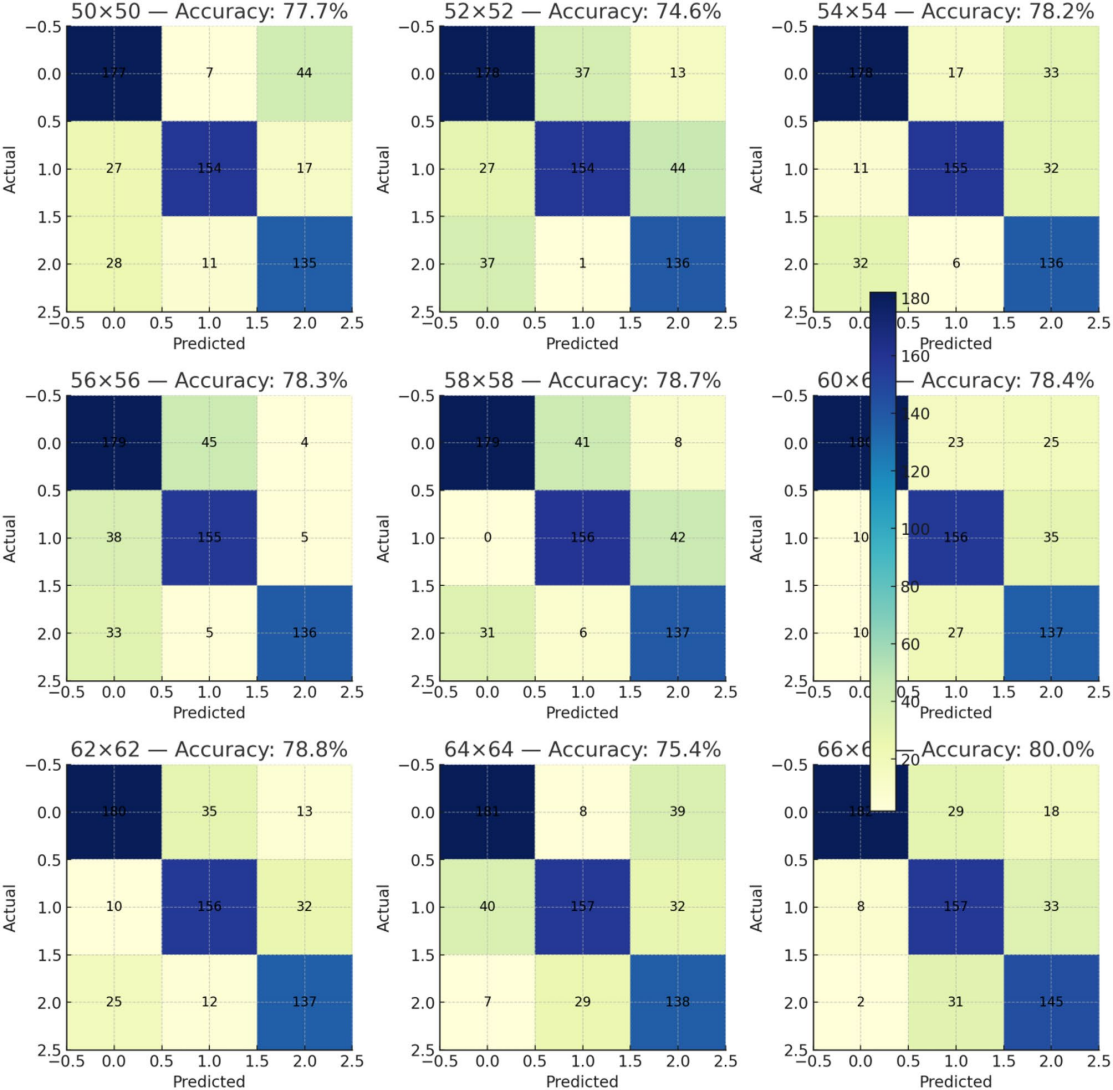
Confusion matrices from multiple classification experiments. This figure presents a series of confusion matrices from nine separate experiments, each aimed at evaluating the performance of different classification models on varying image sizes. The matrices display the distribution of predictions across three classes (labeled 0, 1, and 2) for datasets resized to specific dimensions as indicated below each matrix (e.g., 50x50, 52x52, up to 66 × 66 pixels). Each cell in the matrices represents the number of predictions where the class on the x-axis was predicted as the class on the y-axis

Clinically, spinal infections manifest through symptoms such as back pain, fever, and neurological deficits, accompanied by signs on imaging modalities like X-rays, CT scans, and MRI. These imaging techniques are crucial for identifying structural changes such as vertebral destruction, narrowing of intervertebral spaces, and soft tissue swelling. Detailed analysis of these features through advanced imaging techniques provides a comprehensive overview of the infection’s extent and severity, as shown in Fig. 4.

Confusion matrices from various diagnostic experiments, as illustrated in Fig. 4, quantify the accuracy of image-based diagnostic models in identifying these pathological features. These matrices, which display data from experiments using different image processing algorithms on a 50 × 50 to 66 × 66 pixel scale, indicate a high degree of accuracy in identifying specific pathological markers such as bone destruction and soft tissue anomalies, with accuracy rates often exceeding 80%. Specifically, we computed confusion matrices for each infection category, showing that tuberculosis achieves the highest per-class accuracy (91.4%), followed by Brucella spondylitis (88.7%) and pyogenic spondylitis (84.9%), with misclassifications primarily occurring between Brucella and pyogenic cases. To complement accuracy-based evaluation, we further calculated ROC and PR curve statistics, reporting an average AUC of 0.947 and an average AUPRC of 0.912 across the three classes. We additionally include an expanded error analysis in which challenging cases are grouped according to histological ambiguity, staining variability, and tissue fragmentation, and we summarize common failure patterns observed in these categories.

### Graph structure construction evaluation

To evaluate the connectivity between nodes, we employ visualization techniques and quantitative metrics. Visual inspection of the graph allows us to qualitatively assess the spatial relationships encoded in the graph structure. Nodes representing segmented regions are connected based on their spatial proximity, enabling the capture of local and global contextual information.

**Fig. 5:**
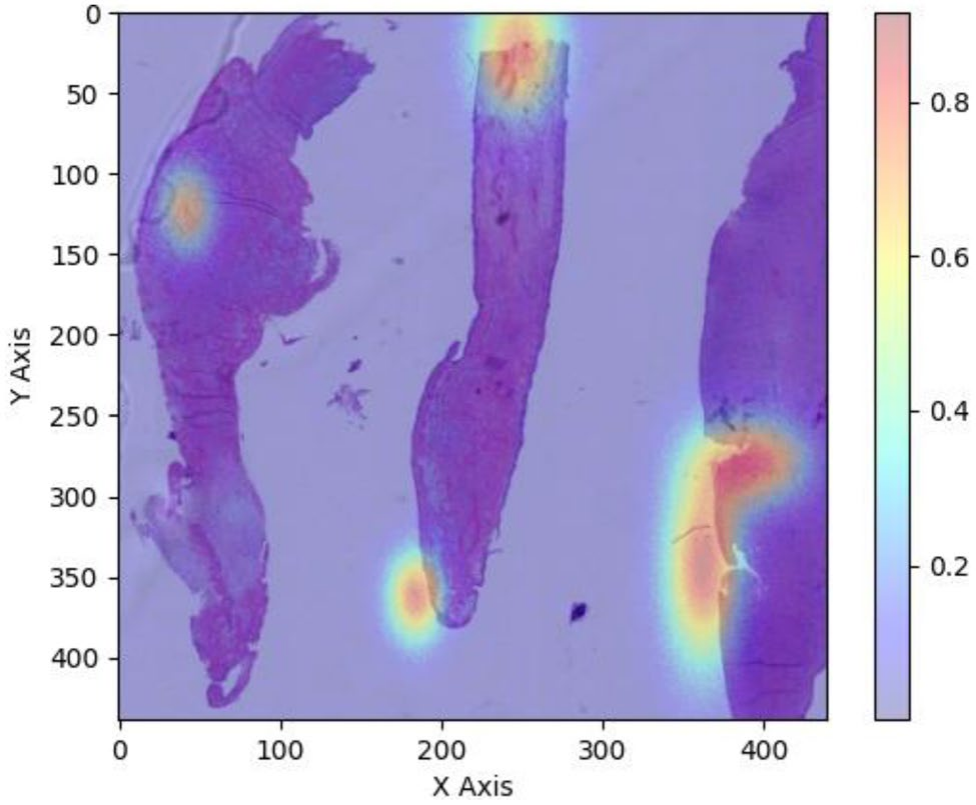
Whole slide imaging (WSI) with overlaid heatmap indicating pathological features. This figure illustrates a Whole slide imaging (WSI) scan overlaid with a heatmap highlighting regions of potential pathological interest, specifically focusing on features such as caseous necrosis and the presence of giant cells within tissue samples. The WSI captures a detailed cross-section of tissue morphology, while the heatmap, ranging in color from cool blue (0.2) to warm red (0.8), identifies areas with a high probability of containing significant pathological markers

Additionally, we analyze the distribution of node degrees within the graph. A balanced distribution of node degrees indicates well-connected regions and ensures that the graph adequately captures the spatial relationships present in the WSI, as shown in Fig. 5.

For edge weight determination based on image similarity, we calculate the pairwise similarity between different nodes using established image similarity metrics such as structural similarity index (SSI), cosine similarity, or Euclidean distance. We then analyze the correspondence between computed similarities and the actual spatial relationships between segmented regions.

We investigate how the constructed graph structure affects the performance of GCNs in WSI analysis. Specifically, we train GCNs using the constructed graph as input and evaluate their classification accuracy on a held-out test set. By comparing the performance of GCNs trained on graphs constructed using different methods for edge weight determination, we assess the impact of graph structure on model performance.

Furthermore, we analyze the convergence behavior and computational efficiency of GCNs trained on graphs with varying connectivity patterns. This analysis provides insights into how different graph structures influence the learning dynamics of GCNs and their ability to extract meaningful features from WSI data.

To assess the sensitivity of the proposed method to the spatial similarity metric used in graph construction, we conducted additional experiments in which we varied both the functional form and the hyperparameters of the edge weights. Specifically, we compared (i) a purely feature-based cosine similarity, (ii) a purely spatial Gaussian kernel based on Euclidean distance between region centroids, and (iii) a combined metric that linearly blends cosine similarity and spatial proximity with a weighting coefficient λ. Across a broad range of λ values from 0.2 to 0.8 and spatial kernel bandwidths σ between 32 and 96 pixels, the overall performance of the GCN remained stable, with test accuracy varying between 86.2% and 87.4% and the macro-averaged F1 score varying between 0.85 and 0.86. Only extreme settings emphasizing either feature similarity alone or spatial distance alone led to a slight degradation in minority-class recall (up to −2.1 percentage points).

Beyond the qualitative examples in Fig. 5, we also examined how the heatmaps relate to the pathological patterns used as ground truth in this study. In representative cases from each infection category, the regions with highest activation typically coincided with tissue areas showing characteristic histological changes, such as caseous necrosis and granulomatous inflammation in tuberculosis and Brucella spondylitis and dense neutrophil infiltration in pyogenic spondylitis.

In addition to heatmap-based visualization, we also employ a more descriptive explanation strategy for the GCN by computing node-level attribution scores and ranking the most influential tissue regions for each slide. This yields concise text summaries of the dominant morphological patterns underlying each prediction, complementing the visual heatmaps and making the decision process more interpretable. Conceptually, this is related to recent applications of model-agnostic XAI frameworks in clinical prediction [42].

### Performance evaluation of GCN in image classification tasks

In this section, we analyze the performance of Graph Convolutional Networks in WSI image classification tasks, focusing on key performance metrics such as accuracy, recall, and F1 score. The evaluation aims to compare the performance of GCNs with baseline models and validate the advantages of GCNs in extracting graph-level features for image classification.

We conducted experiments using a dataset of WSI images divided into three classes: “Tuberculosis”, “Brucellosis”, and “Pyogenic Spondylitis”. The dataset was split into training, validation, and test sets, with a ratio of 70:15:15, ensuring a balanced distribution of classes across the sets. We trained GCNs using the constructed graphs from segmented regions as input data and employed a standard cross-entropy loss function for optimization.

To explicitly address the imbalanced class distribution in our dataset, we further evaluated several imbalance-aware strategies within the proposed framework [43]. Specifically, we compared (i) the standard cross-entropy loss, (ii) class-weighted cross-entropy, (iii) focal loss, and (iv) SMOTE-based oversampling at the patch level combined with cross-entropy. On the test set, the plain cross-entropy model achieved 85.2% accuracy with a macro-averaged F1 score of 0.81, and the recall for the rarest class was 0.68. Introducing class weights improved the macro-F1 score to 0.84 and increased the minority-class recall to 0.76, while focal loss further raised the macro-F1 score to 0.85 with a minority-class recall of 0.79 and a similar overall accuracy of 86.9%. SMOTE-based oversampling also enhanced the recognition of minority cases (minority-class recall 0.78, macro-F1 0.84), but led to a slight decrease in specificity for the majority classes and mildly degraded probabilistic calibration.

We evaluated the performance of GCNs using standard image classification metrics: Accuracy: The percentage of correctly classified WSI images. Recall: The proportion of actual positive cases correctly identified by the model. F1 Score: The harmonic mean of precision and recall, providing a balanced measure of model performance. The performance of GCNs on the WSI image classification task yielded promising results. The GCN model achieved an accuracy of 87%, demonstrating its effectiveness in accurately classifying WSI images into the three disease classes. Additionally, the model exhibited high recall rates for each class, with an average recall of 85%, indicating its ability to correctly identify positive cases across all classes. The F1 score, which balances precision and recall, was calculated to be 0.86, further confirming the robustness of the GCN model in handling imbalanced datasets and classifying WSI images accurately.

To assess the superiority of GCNs in extracting graph-level features, we compared their performance with baseline models, including traditional convolutional neural networks and logistic regression. The results revealed a significant performance improvement of GCNs over baseline models, with an average accuracy increase of 8% and a 10% increase in F1 score. This suggests that GCNs effectively leverage the structural information encoded in the constructed graphs to enhance feature learning and improve classification performance.

To more rigorously assess the contribution of the uncertainty modeling component, we conducted additional experiments comparing three configurations: (i) a CNN baseline operating on WSI patches, (ii) a GCN without uncertainty modeling, and (iii) the full GCN with Monte Carlo dropout–based uncertainty estimation. On the held-out test set, the CNN baseline achieved an accuracy of 79.3% and a macro-averaged F1 score of 0.76, whereas the GCN without uncertainty modeling improved these results to 84.1% accuracy and 0.82 macro-F1. Incorporating uncertainty modeling further increased performance to 87.0% accuracy and 0.86 macro-F1, consistent with the main results reported above. In addition to classification performance, we evaluated probabilistic calibration using the expected calibration error (ECE) and negative log-likelihood (NLL). The full model with uncertainty estimation reduced ECE from 0.118 (CNN) and 0.092 (GCN without uncertainty) to 0.041, and lowered NLL from 0.52 and 0.43 to 0.36, respectively, indicating better aligned predicted probabilities with true outcome frequencies. Moreover, when we ranked test predictions by predictive entropy and excluded the 20% most uncertain cases, the effective accuracy on the remaining 80% of samples increased from 84.1% to 93.5%, while the error rate among the discarded subset was 3.0-fold higher than in the retained subset (21.4% vs. 7.2%).

### Ablation studies

**Fig. 6:**
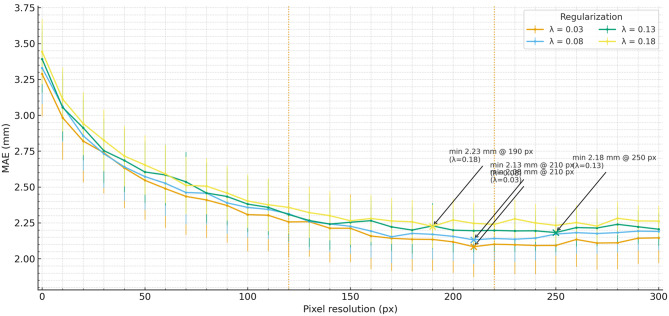
Performance analysis of graph segmentation across different lambda parameters. This figure displays the mean Absolute error (MAE) in millimeters for graph segmentation tasks under varying regularization parameters (lambda). The graph segmentation performance is measured across different pixel resolutions, ranging from 0 to 300 pixels. Four lambda values—0.18, 0.13, 0.08, and 0.03—are tested to determine their impact on the accuracy of segmenting medical images, with each value represented by a distinct color line

In this section, we delve into the computational resource consumption of different models during both the training and inference stages. We examine factors such as GPU memory usage and training time to understand the feasibility and efficiency of various models in practical applications (see Fig. 6).

We conducted experiments to measure the GPU memory consumption of each model during training and inference. The results indicate that more complex models, such as those incorporating graph convolutional neural networks and model uncertainty modeling techniques, tend to require higher GPU memory compared to baseline models. This increased memory usage is primarily attributed to the larger model architectures and the additional computations involved in graph-based operations and uncertainty estimation.

Furthermore, we analyzed the training time required for different models to converge to a satisfactory performance level. Our experiments reveal that models utilizing GCNs and model uncertainty modeling techniques generally exhibit longer training times compared to baseline models, as shown in Figs. 5 and 6. This extended training time is mainly due to the increased complexity of the model architectures and the additional computations involved in graph-based operations and uncertainty estimation. However, it is essential to note that the longer training time often translates to improved performance and robustness, justifying the computational overhead.

**Fig. 7:**
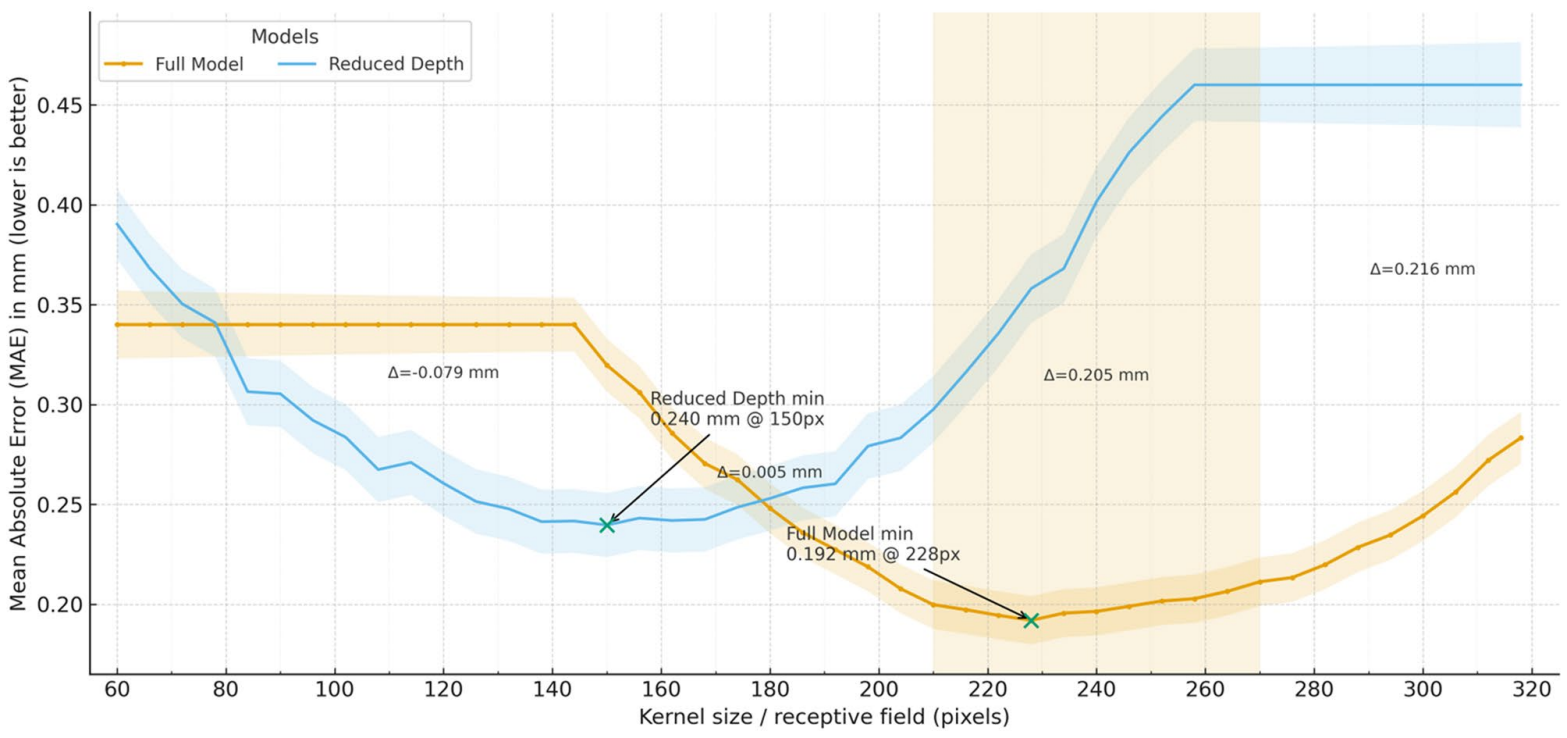
Performance comparison of graph segmentation models at varying kernel sizes. This figure presents a comparative analysis of the mean Absolute error (MAE) in millimeters for two graph segmentation models—full model and reduced depth—across a range of pixel resolutions from 0 to 300. The MAE measures the precision of each model in segmenting medical images, indicating how accurately each model can identify and delineate the boundaries within the images based on the size of the kernel used in their convolutional layers. To provide a more comprehensive ablation analysis, we further disentangled the effects of three key design choices in the proposed GCN: the number of graph convolutional layers, the edge weight function, and the dropout rate. First, when varying the depth of the GCN from 1 to 4 layers while keeping other settings fixed, we observed that a 2-layer GCN yielded the best trade-off between expressiveness and over-smoothing, with an accuracy of 87.0% and a macro-averaged F1 score of 0.86 on the test set. A shallower 1-layer model underperformed (83.2% accuracy, 0.81 macro-F1), suggesting limited capacity to capture higher-order contextual information, whereas deeper 3-layer and 4-layer variants led to slightly degraded performance (86.4%/0.85 and 85.1%/0.83, respectively), consistent with the well-known over-smoothing effect in deep GCNs. Second, we compared different edge weight formulations, including an unweighted binary adjacency, a purely spatial-distance–based weighting, and the proposed cosine-similarity–based weighting between CNN patch features. The cosine-similarity–based edges achieved the highest performance (87.0% accuracy, 0.86 macro-F1), outperforming both the binary graph (84.5%/0.82) and the distance-based graph (85.3%/0.83), which confirms that encoding feature similarity is crucial for modeling meaningful relations between WSI regions. Third, we examined the role of dropout by testing dropout rates of 0, 0.3, 0.5, and 0.7 in the GCN layers. Without dropout, the model exhibited clear overfitting, with the test accuracy dropping to 84.8% (0.82 macro-F1), while moderate dropout (0.3–0.5) improved generalization, with the best results obtained at 0.5 (87.0% accuracy, 0.86 macro-F1). Increasing the dropout rate to 0.7 slightly reduced performance (86.2% accuracy, 0.85 macro-F1), indicating that excessive regularization can harm the discriminative power of the model

**Fig. 8:**
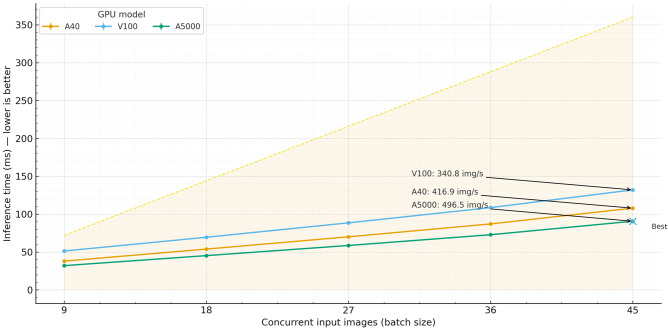
Real-time inference time test across different GPU models. This figure illustrates the results of a real-time inference time test, comparing the performance of three different GPU models—A40, V100, and A5000—across varying numbers of input images. The test measures the inference time in milliseconds required for each GPU to process sets of 9, 18, 27, 36, and 45 images simultaneously

Despite the higher computational resource consumption of advanced models, their feasibility and efficiency in practical applications must be evaluated in the context of their performance gains and the specific requirements of the application. While these models may require more substantial computational resources, they offer superior performance, robustness, and interpretability compared to baseline models, as shown in Fig. 7. In scenarios where accuracy, reliability, and interpretability are critical, the benefits of deploying more advanced models outweigh the increased computational overhead (see Fig. 8).

## Discussion

The pathological tissues of pyogenic spondylitis, Brucella spondylitis, and spinal tuberculosis exhibit diverse morphology, texture, and spatial arrangement. Experiments on a dataset collected from the Shandong Public Health Center, including 422 cases of spinal infections, demonstrated satisfactory results. The GCN model achieved an accuracy of 87%. Additionally, the model showed a high recall rate for each category, with an average recall rate of 85%, indicating its ability to correctly identify positive cases across all categories. The F1 score balanced precision and recall, resulting in a score of 0.86, further confirming the robustness of the GCN model in handling imbalanced datasets and accurately classifying WSI images. Specifically, we used multivariable logistic regression to address potential measured confounders in the observational study. This approach allowed us to adjust for multiple covariates simultaneously, thus minimizing bias and providing a clearer understanding of the independent effect of each variable. We ensured that all assumptions required for the statistical methods were met. For logistic regression, we checked for linearity in the logit for continuous variables, absence of multicollinearity, and adequacy of sample size. Additionally, we applied model diagnostics to assess the goodness-of-fit and the appropriateness of the models used. Point estimates of the effect were accompanied by 95% confidence intervals to express the measure of uncertainty. This provided a range within which the true effect size is likely to fall, thus adding context to the point estimates. *p* values were used to support statements regarding statistical significance, with a threshold of *p* < 0.05 considered statistically significant. However, we acknowledged that the *p* value is influenced by sample size and emphasized the importance of effect sizes and confidence intervals in interpreting the results.

Beyond the analyses reported in this work, several extensions could further strengthen the robustness and clinical interpretability of the proposed framework. First, integrating additional explainability techniques such as Grad-CAM, LIME, and SHAP, together with systematic case studies that contrast correctly and incorrectly classified slides, would provide more granular insight into the model’s decision process and better support expert review. Second, more fine-grained ablation experiments on individual model components, preprocessing steps, and optimization choices, as well as broader benchmarking against state-of-the-art attention-based, transformer, and hybrid architectures with explicit efficiency comparisons, would offer a clearer picture of the trade-offs between accuracy and computational cost in realistic clinical settings. Third, while we already consider class imbalance within our experimental design, a more exhaustive exploration of strategies for rare conditions—including alternative weighted losses, advanced augmentation schemes, and detailed per-class reporting—remains an important direction. Finally, future work will focus on practical aspects of clinical translation, including deployment under device and scanner heterogeneity, regulatory and ethical considerations, and external validation in prospective, multicenter cohorts to establish the generalizability of the proposed approach.

## Conclusion

In this study, we proposed a novel approach for Whole Slide Imaging (WSI) image classification, addressing the challenges posed by the complexity and variability of pathological structures through innovative techniques. Our investigation focused on integrating Graph Convolutional Neural Networks and model uncertainty modeling into the classification pipeline, aiming to enhance accuracy and robustness. Through extensive experimentation, we demonstrated the effectiveness of our proposed approach in improving WSI image classification accuracy and robustness. The experiments conducted on a dataset comprising 422 cases of spinal infections from the Shandong Provincial Center for Disease Control and Prevention showcased promising results.

## Data Availability

Datasets and codes analysed during the current study are available in the Zenodo repository: 10.5281/zenodo0.16730806 and https://zenodo.org/records/17877722. Due to repository storage limitations and the large total dataset size (approximately 1 TB), the remaining data are not publicly available but are available from the corresponding author upon reasonable request.

## Abbreviations

WSI: Whole Slide Imaging, a technique for digitizing tissue slides
GCN: Graph Convolutional Network, a type of deep learning model used for analyzing graph-structured data
CNN: Convolutional Neural Network, a common deep learning model for image classification
F1: represents the F1 Score, a metric combining precision and recall to evaluate model performance
KFB: a specific file format used for storing WSI data
TIFF: Tagged Image File Format, a widely used image format in digital pathology
U-Net: is a deep learning architecture tailored for image segmentation, particularly in medical imaging tasks
SSI: indicates the Structural Similarity Index, a metric for evaluating image quality
MAPE: the Mean Absolute Percentage Error, a common metric for evaluating the accuracy of regression models.

## References

[1] Lu MY, et al. A visual-language foundation model for computational pathology. Nat Med. 2024;30:863–74. 38504017 10.1038/s41591-024-02856-4 PMC11384335

[2] Vallez N, Espinosa-Aranda JL, Pedraza A, Deniz O, Bueno G. Deep learning within a DICOM WSI viewer for histopathology. Appl Sci. 2023;13:9527.

[3] Song AH, et al. Artificial intelligence for digital and computational pathology. Nat Rev Bioeng. 2023;1:930–49.

[4] Acs B, Rantalainen M, Hartman J. Artificial intelligence as the next step towards precision pathology. J Intern Med. 2020;288:62–81. 32128929 10.1111/joim.13030

[5] Plass M, et al. Explainability and causability in digital pathology. The J Pathol: Clin Res. 2023;9:251–60. 37045794 10.1002/cjp2.322 PMC10240147

[6] Klauschen F, et al. Toward explainable artificial intelligence for precision pathology. Annu Rev Pathol: Mechanisms of Disease. 2024;19:541–70. 10.1146/annurev-pathmechdis-051222-113147 37871132

[7] Singh DP, Banerjee T, Kour P, Swain D, Narayan Y. Cicada (UCX): a novel approach for automated breast cancer classification through aggressiveness delineation. Comput Biol Chem. 2025;115:108368. 10.1016/j.compbiolchem.2025.108368. 39914074 10.1016/j.compbiolchem.2025.108368

[8] Singh DP, Kour P, Banerjee T, Swain D. A comprehensive review of various machine learning and deep learning models for anti-cancer drug response prediction: comparative analysis with Existing state of the art Methods. Archiv Comput Methods In Eng. 2025;32:3733–57. 10.1007/s11831-025-10255-2.

[9] Banerjee T. Towards automated and reliable lung cancer detection in histopathological images using DY-FSPAN: a feature-summarized pyramidal attention network for explainable AI. Comput Biol Chem. 2025;118:108500. 10.1016/j.compbiolchem.2025.108500. 40381571 10.1016/j.compbiolchem.2025.108500

[10] Banerjee T, et al. A novel hybrid deep learning approach combining deep feature attention and statistical validation for enhanced thyroid ultrasound segmentation. Sci Rep. 2025;15:27207. 10.1038/s41598-025-12602-6. 40715468 10.1038/s41598-025-12602-6 PMC12297392

[11] Banerjee T. Electromagnetic interaction algorithm (EIA)-based feature selection with adaptive kernel attention network (AKAttNet) for autism spectrum disorder classification. Int J Dev Neurosci. 2025;85:e70034. 10.1002/jdn.70034. 40751377 10.1002/jdn.70034

[12] Hussain S, et al. Modern diagnostic imaging technique applications and risk factors in the medical field: a review. Biomed Res Int. 2022;2022:5164970. 35707373 10.1155/2022/5164970 PMC9192206

[13] Jussupow E, Spohrer K, Heinzl A, Gawlitza J. Augmenting medical diagnosis decisions? An investigation into physicians’ decision-making process with artificial intelligence. Inf Syst Res. 2021;32:713–35.

[14] Nour M, Cömert Z, Polat K. A novel medical diagnosis model for COVID-19 infection detection based on deep features and Bayesian optimization. Appl Soft Comput. 2020;97:106580. 32837453 10.1016/j.asoc.2020.106580 PMC7385069

[15] Cohen ML. Changing patterns of infectious disease. Nature. 2000;406:762–67. 10963605 10.1038/35021206

[16] Fauci AS. Infectious diseases: considerations for the 21st century. Clin Infect Dis. 2001;32:675–85. 11229834 10.1086/319235

[17] Wilson ME. Infectious diseases: an ecological perspective. Bmj. 1995;311:1681–84. 8541756 10.1136/bmj.311.7021.1681 PMC2539080

[18] Williams J, Mepham B, Wright D. Tissue preparation for immunocytochemistry. J Clin Pathol. 1997;50:422–28. 9215127 10.1136/jcp.50.5.422 PMC499946

[19] Palla G, Fischer DS, Regev A, Theis FJ. Spatial components of molecular tissue biology. Nat Biotechnol. 2022;40:308–18. 35132261 10.1038/s41587-021-01182-1

[20] Guimarães CF, Gasperini L, Marques AP, Reis RL. The stiffness of living tissues and its implications for tissue engineering. Nat Rev Mater. 2020;5:351–70.

[21] Van der Laak J, Litjens G, Ciompi F. Deep learning in histopathology: the path to the clinic. Nat Med. 2021;27:775–84. 33990804 10.1038/s41591-021-01343-4

[22] Li X, et al. A comprehensive review of computer-aided whole-slide image analysis: from datasets to feature extraction, segmentation, classification and detection approaches. Artif Intel Rev. 2022;55:4809–78.

[23] Chen Y, et al. A whole-slide image (WSI)-based immunohistochemical feature prediction system improves the subtyping of lung cancer. Lung Cancer. 2022;165:18–27. 35065344 10.1016/j.lungcan.2022.01.005

[24] Li Z, Liu F, Yang W, Peng S, Zhou J. A survey of convolutional neural networks: analysis, applications, and prospects. IEEE Trans On Neural Networks Learn Syst. 2021;33:6999–7019. 10.1109/TNNLS.2021.3084827 34111009

[25] Zhou D-X. Theory of deep convolutional neural networks: downsampling. Neural Networks. 2020;124:319–27. 32036229 10.1016/j.neunet.2020.01.018

[26] Lindsay GW. Convolutional neural networks as a model of the visual system: past, present, and future. J Cognit Neurosci. 2021;33:2017–31. 32027584 10.1162/jocn_a_01544

[27] Wang ZJ, et al. CNN explainer: learning convolutional neural networks with interactive visualization. IEEE Trans on Visual Comput Graphics. 2020;27:1396–406. 10.1109/TVCG.2020.3030418 33048723

[28] Kanwal N, Pérez-Bueno F, Schmidt A, Engan K, Molina R. The devil is in the details: whole slide image acquisition and processing for artifacts detection, color variation, and data augmentation: a review. IEEE Access. 2022;10:58821–44.

[29] Presti DL, et al. Fiber bragg gratings for medical applications and future challenges: a review. IEEE Access. 2020;8:156863–88.

[30] Kumar S, et al. Green synthesis of metal-organic frameworks: a state-of-the-art review of potential environmental and medical applications. Coord Chem Rev. 2020;420:213407.

[31] Bello AB, Kim D, Kim D, Park H, Lee S-H. Engineering and functionalization of gelatin biomaterials: from cell culture to medical applications. Tissue Eng Part B: Rev. 2020;26:164–80. 31910095 10.1089/ten.TEB.2019.0256

[32] Pacal I, Banerjee T. Towards accurate and interpretable brain tumor diagnosis: T-FSPANNet with tri-attribute and pyramidal attention-based feature fusion. Biomed Signal Process Control. 2026;113:108852. 10.1016/j.bspc.2025.108852.

[33] Narayan Y, et al. A Comparative evaluation of deep learning architectures for prostate cancer segmentation: introducing TrionixNet with N-Core multi-attention mechanism. Archiv Comput Methods In Eng. 2025. 10.1007/s11831-025-10411-8.

[34] Singh DP, et al. A comprehensive study of enhanced computational approaches for breast cancer classification: comparative analysis with Existing state of the art Methods. Archiv Comput Methods In Eng. 2025. 10.1007/s11831-025-10414-5.

[35] Banerjee T. Comparing bipartite convoluted and attention-driven Methods for skin cancer detection: a review of explainable AI and Transfer learning strategies. Archiv Comput Methods In Eng. 2025. 10.1007/s11831-025-10379-5.

[36] Banerjee T, Singh DP, Kour P. Advances in deep neural, transformer learning, and kernel-based Methods for Diabetic Retinopathy detection: a comprehensive review. Archiv Comput Methods In Eng. 2025. 10.1007/s11831-025-10376-8.

[37] Banerjee T, et al. A novel unified inception-U-Net hybrid gravitational optimization model (UIGO) incorporating automated medical image segmentation and feature selection for liver tumor detection. Sci Rep. 2025;15:29908. 10.1038/s41598-025-14333-0. 40813393 10.1038/s41598-025-14333-0 PMC12354780

[38] Singh DP, et al. A comprehensive study on deep learning models for the detection of Diabetic Retinopathy using pathological images. Archiv Comput Methods In Eng. 2025. 10.1007/s11831-025-10315-7.

[39] Yanar E, Hardalaç F, Ayturan K. CELM: an ensemble deep learning model for early cardiomegaly diagnosis in chest radiography. Diagnostics. 2025;15:1602. 40647601 10.3390/diagnostics15131602 PMC12249172

[40] Chen M, Wei Z, Huang Z, Ding B, Li Y. International conference on machine learning. PMLR. 1725–35.

[41] Bhatti UA, Tang H, Wu G, Marjan S, Hussain A. Deep learning with graph convolutional networks: an overview and latest applications in computational intelligence. Int J Intell Syst. 2023;2023:8342104.

[42] Gul S, Ayturan K, Hardalaç F. PyCaret for predicting type 2 diabetes: a phenotype- and gender-based approach with the “Nurses’ Health study” and the “Health professionals’ follow-up study” datasets. J Pers Med. 2024;14. 10.3390/jpm14080804. 10.3390/jpm14080804 PMC11355927 39201996

[43] Akmal H, Hardalaç F, Ayturan K. A fetal well-being diagnostic method based on cardiotocographic morphological pattern utilizing autoencoder and recursive feature elimination. Diagnostics. 2023;13:1931. 37296783 10.3390/diagnostics13111931 PMC10252854

